# Modelling gene expression profiles related to prostate tumor progression using binary states

**DOI:** 10.1186/1742-4682-10-37

**Published:** 2013-05-31

**Authors:** Emmanuel Martinez, Victor Trevino

**Affiliations:** 1 Tecnológico de Monterrey, Campus Monterrey, Cátedra de Bioinformática, Monterrey, Nuevo León 64849, México

## Abstract

**Background:**

Cancer is a complex disease commonly characterized by the disrupted activity of several cancer-related genes such as oncogenes and tumor-suppressor genes. Previous studies suggest that the process of tumor progression to malignancy is dynamic and can be traced by changes in gene expression. Despite the enormous efforts made for differential expression detection and biomarker discovery, few methods have been designed to model the gene expression level to tumor stage during malignancy progression. Such models could help us understand the dynamics and simplify or reveal the complexity of tumor progression.

**Methods:**

We have modeled an on-off state of gene activation per sample then per stage to select gene expression profiles associated to tumor progression. The selection is guided by statistical significance of profiles based on random permutated datasets.

**Results:**

We show that our method identifies expected profiles corresponding to oncogenes and tumor suppressor genes in a prostate tumor progression dataset. Comparisons with other methods support our findings and indicate that a considerable proportion of significant profiles is not found by other statistical tests commonly used to detect differential expression between tumor stages nor found by other tailored methods. Ontology and pathway analysis concurred with these findings.

**Conclusions:**

Results suggest that our methodology may be a valuable tool to study tumor malignancy progression, which might reveal novel cancer therapies.

## Background

Cancer is a complex and multi-factorial disease. Hanahan and Weinberg define the hallmarks of cancer as the manifestation of alterations in cell physiology, including limitless of replicative potential, sustained angiogenesis, evasion of apoptosis, self-sufficiency of growth signals, insensitivity to antigrowth signals, tissue invasion and metastasis [1]. The order and mechanisms in which these alterations emerge during malignancy progression is thought to vary between individuals and tumor types [1]. Moreover, studies have proven that cancer is a genetic disease [2] which is characterized by mutations in several cancer-related genes such as oncogenes, tumor-suppressor genes and stability genes [3]. The diversity and interconnection of these factors and mutations makes tumor progression difficult to model, study, and predict.

Studies have shown that tumors are heterogeneous in mutations and gene expression during progression to malignancy [4,5]. The consequence of alterations in oncogenes or their regulators is the constitutive activation compared to wild-type gene. The activity of tumor-suppressor genes (TSG) is affected in the opposite way; disruptions lead in function degradation. In addition to oncogenes and TSG, stability genes or caretakers when mutated promote tumorigenesis by decreasing the restoration of DNA replication mistakes or by the inability to correct all mutations when cells have been exposed to mutagens [2].

Microarray technology for gene expression profiling has proven to be successful in a variety of experimental settings [6,7] having the potential to discover the diversified and dynamic molecular states during tumor progression. In malignancy progression, it has shown that increases or decreases in activity can be traced by changes in gene expression [5]. The analysis of microarray data is, nevertheless, complex; the results are dependent on the analysis method and noise handling generating ambiguous or complementary results [8]. Besides the microarray data inherent problems, the examination of tumor progression is complicated by the limitation of the sampling time typically performed at diagnosis and by a staging system mainly based on phenotypical features [9]. This raises the issue that cancer samples may be labeled under the same stage regardless of their molecular state. In addition, there are few datasets designed to study tumor progression. Therefore, tools that analyze gene expression by novel approaches are needed and appreciated by medical, biological and scientific community.

Despite the massive efforts made to detect differential expression and biomarkers, few methods have been designed to model the gene expression level to tumor stage during malignancy progression. Such models could help us understand the dynamics and simplify or reveal the complexity of tumor progression. For example, in breast cancer, low and high grades have been in addition divided into six molecular subtypes using principal component analysis followed by a tailored clustering method [10], and gene co-expression networks have been used to form subgroups of different relapse-free survival times [11]. In other cancers, simple differential gene expression combined with enrichment analysis [12] has been used to obtain common transcriptional profiles shared between cancers of several tissues [13], which was further expanded to allow combinations and extensions of experimentally-designed sets of genes to uncover molecular concepts during prostate cancer progression [5]. Recently, other methods have been applied to tumor progression, such as significant minimum spanning trees among clusters of co-expressed genes [14], genes over-expressed between the first and the last tumor stages [15], over-represented pathways of differentially expressed genes between progression stages [16], and temporal re-ordering of samples to by genes of minimum expression changes along progression [17].

In this paper, we contribute a novel yet simple approach to study tumor progression assuming tumor heterogeneity. We propose a method to identify relevant genes related to tumor progression transforming the distribution of gene expression to binary states per sample then modeling the distribution of sample states within a progression stage to assign also a binary state. We believe that this approach is, to some extent, robust to tumor heterogeneity and noise. Our results in two prostate cancer datasets show that significant genes resemble the ideal profiles of oncogenes and TSG during tumor malignancy progression and that a large number of genes were not found in the original publication neither using well-known differential expression methods.

## Methods

### Binary states model (BSM)

**Figure 1:**
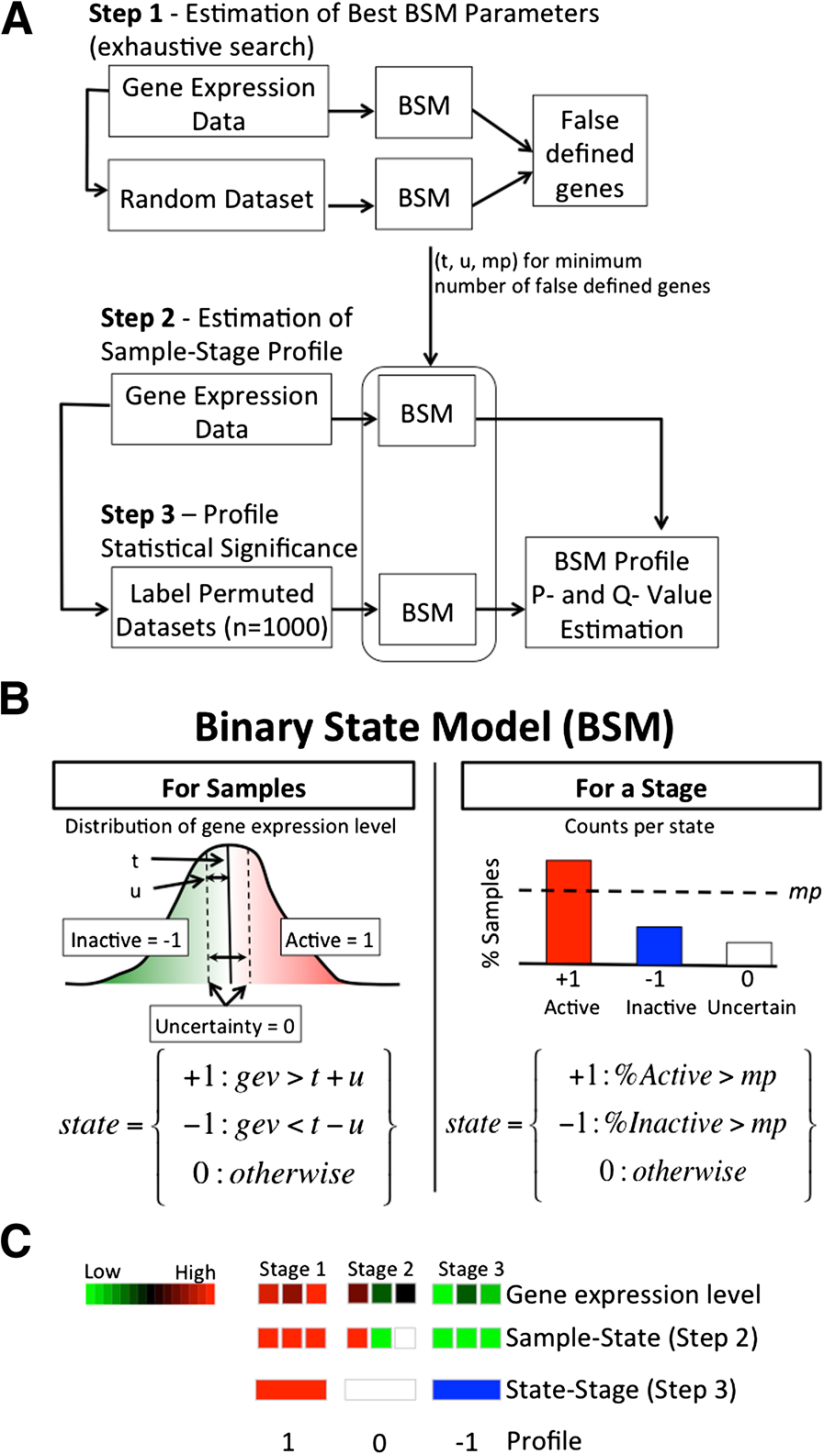
**Binary state model algorithm.** (**A**) Overall methodology from gene expression to gene selection. First, an exhaustive search is used for parameter estimation of the binary state model. Then, *mp*, *t* and *u* parameters found are used to estimate sample and stage states in the dataset and in its permutated versions needed to estimate a stage-state profile null distribution. Gene selection is based on FDR of state-stage profiles. (**B**) Binary state model for samples and stages. *gev* stands for gene expression value. (**C**) Graphical example of the algorithm. Squares and rectangles represent samples and stages respectively.

The overall methodology (Figure 1A) is based on binary states first to individual samples then to tumor stages (Figure 1B). For individual samples, we generate ideal binary states representing whether a gene is active (value=1) or inactive (value=−1). In addition, we assign a value of 0 when the state for a sample cannot be determined. We hypothesized that the normalized intensity of a gene in a sample can be either above, below or within an uncertainty zone defining the gene as active=1, inactive=−1, or uncertain=0 respectively. The uncertainty region is centered in *t* and limited by *t*-*u* and *t*+*u*, where the parameters *t* and *u* represents the cut-off and uncertainty respectively. Some authors have used similar approaches [18-21]. Then; we defined the state per stage as active=1 or inactive=−1 when the proportion of samples within that stage and state is higher or equal than a proportion parameter, *mp*. The stage-state can also be designed as uncertain=0 when it could not be assigned as active nor inactive. Next, we simply concatenate the gene state per stage to generate a profile of 1’s, 0’s, and -1’s separated by period for representation. For example, the profile 1.0.-1 would represent that the gene is active in the first stage, undefined in the second stage and inactive in the last stage (Figure 1C). For a textual description of the algorithm and pseudo-code, see supplementary data.

### Parameters estimation

To find the best *t*, *u*, and *mp* parameters (shown in Figure 1B), we used an exhaustive search of discrete values comparing observed and bootstrap estimations. For the proportion parameter *mp* we used 0.5, 0.6, 0.7, and 0.8 representing 50% to 80% of the samples in the same state. Lower values would generate ambiguity, and higher values would be highly stringent. Similarly, for the cut-off value *t*, we used 0.3, 0.4, 0.5, 0.6, and 0.7. For the uncertainty value *u*, we used 0, 0.25, 0.5, 0.75, and 1 multiplied by the standard deviation of the dataset to adapt the observed variation per gene and fairly compare genes in the same stage. To determine the best of the 100 value combinations of these parameters, we generated artificial datasets composed by uniform distributed random values between 0 and 1 . We used at least *P*=100 random datasets (results for *P*=*1*,*000* and *P*=*10*,*000* yields the same results, so we used 100 for speeding up the pipeline for final users) and ran each set of parameters on each random dataset. Then, for each gene *i* in each random dataset *p*, we set *d*_*ip*_ equal to the number of stages that it was defined as active or inactive (thus not considering uncertainties). Next, for each possible number of stages *s*, from 1 to the total number of tumor progression stages *Z*, we counted the number of genes that were active or inactive in exactly *s* stages, *D*_*sp*_ = *count*(*d*_*ip*_=*s*) and average among all datasets as *DR*_*s*_ = *sum*(*D*_*sp*_) / *P*. *DR*_*s*_ gives an estimation of the number of genes false assigned as active or inactive to *s* stages. Next, we defined the ratio of the cumulative number of false defined genes in at least *s* stages as *F*_*s*_=(*D*_*s*_+*D*_*s*+*1*_+…+*D*_*Z*_)/ (*DR*_*s*_+*DR*_*s*+*1*_+…+*DR*_*Z*_), from *s*=*1*…*Z* where *D*_*k*_ is the observed number of genes defined as active or inactive for *k* stages in the original dataset. Finally, we estimated the total number of non-false assigned genes by *NF*=(*1*-*F*_*1*_)*D_*1*_+(*1*-*F*_*2*_)*D_*2*_+…+(*1*-*F*_*z*_)*D_*z*_. The combination of parameters that yield highest *NF* was then chosen.

### Estimation of stage-state profile significance

Using the best parameter combination, we calculated an empirical *p*-*value* to rank the gene profiles using permutated stage labels such as in SAM [22]. We ran BSM at least 1,000 times to draw the expected probability of each profile by chance. *p*-*values* are calculated dividing 1+the number of times of each profile is found in the permutated dataset by the number of genes of all permutations. We assume that the profiles not frequently found in the permuted datasets are the most significant. *p*-*values* were adjusted using a false discovery rate method to generate *q*-*values*[23,24], which help to finally select genes with statistical significant state-stage profile.

### Simulations on synthetic data

To explore the potential of BSM to identify genes with specific properties for cancer progression, we performed a tailored simulation study generating a dataset containing synthetic gene expression following specific stage-state profiles. To simplify the analysis, we included 2, 3, or 4 stages with 10, 20, 30, 40, or 50 samples each. The range of gene expression was from 0 to 1. To generate active stage-state values (+1), we used Gaussian random numbers whose mean was randomly chosen from .625, 0.750, and 0.875. The standard deviation was randomly chosen from 0.125, 0.250, and 0.375. Only combinations where the *mean* – *sd* >= *0*.*5* were used to ensure an activation level. Similarly, for inactive stage-states (−1), the mean was chosen from 0.125, 0.250, and 0.375, using the same standard deviations and constrained to *mean* + *sd* <= *0*.*5*. For states=0, two normal distributions were used, half of the samples are generated with mean=0.75 and the remaining with mean=0.25, both with sd=0.15. Synthetic datasets included 60 positive synthetic genes for 2 stages, 180 for 3 stages, and 200 for 4 stages, using “ideal” oncogene and TSG profiles (e.g. in 4 stages: *1*.*1*.*1*.-*1*, -*1*.*1*.*1*.-*1*, -*1*.-*1*.-*1*.*1*, *1*.-*1*.-*1*.*1*, *1*.-*1*.-*1*.-*1*, -*1*.*1*.*1*.*1*). A heat map representation of synthetic genes is shown in Additional file 1: Figure S1. Synthetic datasets also include around 4,800 negative synthetic genes (up to 5000) from profiles that do not represent “interesting” state-states (those that have no transitions between 1 and −1, e.g. *1*.*1*.*1*.*1*, -*1*.-*1*.-*1*.*0*, *1*.*0*.*1*.*1*, *0*.-*1*.*0*.-*1*).

### Prostate datasets

We used a prostate cancer dataset available in GEO database as GSE6099 [5,25]. This dataset consists of 20,000 genes in 104 cDNA samples distributed in the following stages ordered by tumor progression: 39 Normal, 13 Prostatic intraepithelial neoplasia (PIN), 32 Prostate cancer (PCA), and 20 Metastases (Met). This dataset was pre-processed from the original raw files using bioconductor [26]. Finally, we uniformized the gene expression values in each sample to values between 0 and 1 in order to eventually compare results from different datasets and technologies. Results with and without uniformization did not change the results of BSM (see Additional file 2: Table S9). The uniformization is performed by changing gene expression values to its corresponding quantile for each sample. We also used the Memorial Sloan-Kettering Cancer Center database of prostate cancer that included 179 prostate samples along four stages (29 normal, 78 Gleason score 5 or 6, 53 Gleason score 7 to 9, and 19 metastasis) [27].

### Comparisons with other methods

To determine whether BSM selects similar genes than those selected by other methods, we estimated the degree of overlap from the genes selected by our method to those selected by commonly used methods such as using t-test [13], wilcoxon-test and f-test [28], cancer outlier profile analysis (COPA) and outlier sum [29,30], SAM [22], and molecular concepts [5]. For t-test and Wilcoxon-test, a comparison of one stage versus all other stages was performed. For COPA, we used the maximum of the quantiles at 75%, 90%, and 95% per stage and took the maximum value. To perform fair comparisons with our method, we used the 215 most significant genes (as those selected by BSM, see results) in all test regardless of the *p*- and *q*-*values*. For simulations, we used the top number of genes equal to the positive synthetic genes.

### Ontology enrichment

Results using BSM and SAM were tested for enrichment for Gene Ontology terms and KEGG pathways using WebGestalt (Duncan, et al. 2010). To highlight differences, we used a 20% FDR as cut-off to select significant enrichment.

## Results and discussion

### Simulation using synthetic datasets

We compared BSM, SAM, f-test, Wilcoxon, COPA/OSUM, and t-test gene selection methods for the synthetic dataset. We ran 40 simulations containing 2, 3, and 4 stages compromising between 10 and 50 samples per stage (Additional file 2: Table S1). Overall, BSM recovered 82% of the 5,720 positive synthetic genes contained in the 40 simulations, SAM, f-test, Wilcoxon, COPA/OSUM, and t-test recovered 69%, 45%, 36%, 7%, and 33% respectively. The BSM performance was 71%, 84%, and 85% for 2, 3, and 4 stages respectively. Overall, BSM recovered 4,701 out of 5,720 genes, including 1,054 genes (26%) that SAM did not find. BSM recovered more genes than SAM in 33 of the 40 simulations. SAM surpassed BSM only in 6 simulations. From these, 4 were two-stages and 3 contained only 10 samples per stage. Although the BSM performance decreases for two stages or for a small number of samples per stage, the results suggest that BSM recover more genes than SAM for idealized profiles related to tumor progression. Therefore, BSM is a valuable tool that can be used in addition to SAM to study tumor progression.

### Prostate cancer dataset

#### Parameter estimation

Our proposal is to model binary states profiles similar to those expected in TSG and oncogene profiles. For this, we first binarized the gene expression to define whether a gene is active (value=1), inactive (value=−1), or uncertain (value=0) as shown in Figure 1. Then, we determined the gene state per stage by determining whether the highest proportion of their samples are active or inactive and higher to a minimum proportion parameter, *mp*. The result is a gene stage-state profile of 1’s, 0’s, and -1’s, which we separate by dots, representing whether the gene in the stage is in summary active, inactive, or uncertain. We estimated the best discrete parameters combinations using bootstrap techniques based on the maximum number of genes correctly assigned to a state. The best combination of parameters found in step 1 were *t*=*0*.*5*, *u*=*0*, and *mp*=*0*.*7* (Additional file 2: Table S2) where 89% of the genes (17,760) were assigned as active or inactive in at least one stage independently whether its profile was significant. Only 2,239 genes (11%) could not be assigned to an activation or inactivation state in any of the four tumor progression stages corresponding to the profile 0.0.0.0. We observed 72 out of the 81 possible stage-states profiles in the Tomlins *et al*. dataset (Additional file 2: Table S3). The distribution of state-stages profiles supports that a diverse set of molecular states exists in tumor progression.

### *p*-*value* estimation

The distribution of state-stages profiles in the permutated dataset is shown in Additional file 2: Table S3. The majority of the profiles were favored to complete inactivation (12%), activation (12%), or uncertain (11%) corresponding to profiles −1.-1.-1.-1, 1.1.1.1 and 0.0.0.0 respectively. We observed that only the 0.24% of the profiles included a transition from active to inactive or from inactive to active of the 50 possible profiles with one transition. However, some transitions were more frequent than others. Two transitions were even more rare in the permutated datasets; only 0.013% of genes in the permutated dataset contained any of the 14 possible profiles with two transitions. These observations indicate that state-stage profiles containing at least one transition are quite rare in permutated dataset and therefore highly significant if observed in tissues. We used this distribution to assign a *p*-*value* for each gene in the Tomlins *et al*. dataset counting the number of times a specific profile was obtained in the permutated dataset divided by the total number of genes times the number of permutations. This *p*-*value* was then corrected using a false discovery rate approach [24]. The results are shown in Table 1 and Additional file 2: Table S3 and discussed in following sections.

**Table 1:** Comparison of genes selected by different methods

	**t-Test**	**f-Test**	**Wilcox**	**Copa**	**SAM**	**Tomlins**	**Barcode**
t-Test	-						
f-Test	0.33	-					
Wilcoxon	0.36	0.47	-				
Copa	0.01	0.01	0.06	-			
SAM	0.36	0.90	0.47	0.01	-		
BSM	0.14	0.26	0.20	0.00	0.28	0.05	0.07-0.37

### Profile distribution

**Figure 2:**
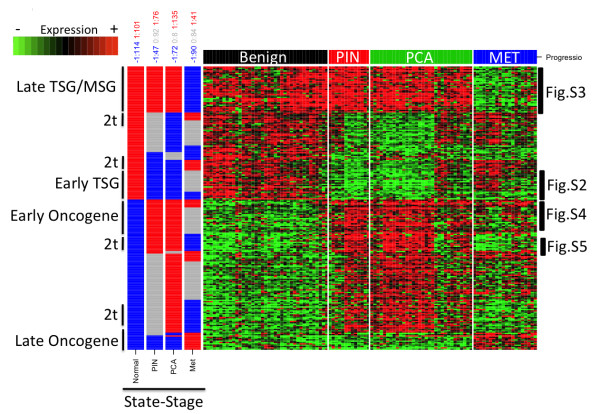
**State**-**Stage representation of significantly selected genes using BSM.** State-Stages are represented as active=1 in red, inactive=−1 in blue, or uncertain=0 in gray. Stages are indicated. Samples are shown in columns whereas genes are ordered by stage-state profile in vertical. Expression values ranges from 0 to 1 corresponding to various levels of colors from green to black then to red. Rank given by SAM is shown for comparison (black represents ranks from 1 to 215, dark gray to 500, light gray to 1000, and white >1000).

From the 20,000 genes, there were 4,970 where the four progression stages used were assigned (none 0 in the profile). Nevertheless, 4,880 were always active (1.1.1.1) or inactive (−1.-1.-1.-1) in the four stages representing ‘flat’ and uninteresting profiles. The others 90 genes had transitions from −1 to 1 or from 1 to −1. We observed that 12,790 of the genes have at least one uncertainty value in their stage-state profile. Uncertainties were more present in PIN and MET stages where the number of samples is small (13 and 20 respectively). These uncertainties may occur in three scenarios, ‘flat’ when the uncertainty is preceded and followed by the same state-stage value (−1.0.-1 or 1.0.1), ‘transitory’ when it is preceded and followed by different state-stage values (−1.0.1 or 1.0.-1), and when the uncertainty is present in the first or last stage (0.x.x.x or x.x.x.0 where x represent any state). All scenarios were highly present, mainly those ‘flat’ and starting or ending with uncertainty (Additional file 2: Table S3). Nevertheless, within the 215 significant genes (Figure 2) the first ‘flat’ uncertain scenario was poorly observed (10 genes for −1.0.-1.1, -1.1.0.1, and 1.-1.0.-1) whereas the second ‘transitory’ scenario was quite common (90 genes from 6 profiles). For the third scenario (starting or ending in 0), we observed 4 profiles for 84 genes only in metastases. These results from uncertainties may indicate a mixed or transitory state between previous and following stages supporting the fact that tumors are heterogeneous within stages and even within individuals [31]. This is also consistent with *in*-*situ* studies showing that markers are commonly present only in a fraction of samples of the same tumor grade [32]. Other studies have shown that 12 and 9 genes on average were mutated in individual breast and colorectal cancers from a total of 122 and 69 genes respectively that were mutated in 11 tumors [4] and reviews of further studies show that between 33 to 66 genes are mutated in several common cancers [33]. Given the assumption of this mutational heterogeneity found in those studies (33 to 66 genes), changes in gene expression from these genes, or more importantly those they control directly or indirectly, are expected to be also altered and rather heterogeneous. This is consistent with the observed heterogeneity patterns we found.

### Significant stage-state profiles

**Figure 3:**
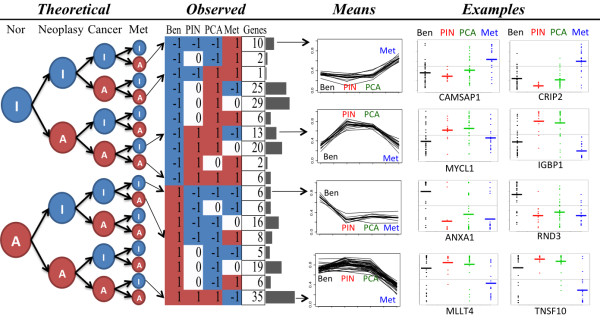
**Theoretical and observed profiles and examples.** A and I represent active or inactive gene expression respectively. All possible state paths along progression are shown. Gene expression is shown in vertical axis in *Means* and *Examples*. Line in *Means* represent a gene average expression. Dots in *Examples* represent samples and their average by a horizontal line.

Using a *q*-*value* of 0.2 equivalent to *p*-*values* between 1.1e-5 and 5e-8, 215 of the 20,000 genes profiles were significant in Tomlins *et al*. dataset (Figure 2 and 3 and Table 2). The list of the selected genes is shown in Additional file 2: Table S4. All profiles involved at least one transition. From the theoretical defined stage-states (Figure 3, left panel), we observed 7 out of the 14 progression-interesting profiles (marked with arrows in Figure 3) representing 79 genes (37%). So, 11 out of the 18 significant profiles, representing 136 genes (63%), have at least one uncertainty state. State-stage profiles similar to oncogenes and TSG are clearly observed and well supported by specific gene examples (Figure 3 and marked in Figure 2). We observed diverse patterns corresponding to TSG profiles starting with an activation followed by deactivations in PIN (1.-1.-1.-1, 1.-1.-1.0, 1.-1.0.-1, 28 genes) or MET (1.1.1.-1, 35 genes) marked as early and late TSG in Figure 2 respectively. We also found TSG profiles where the stage-state is inactive in PCA but only when uncertain in PIN (1.0.-1.-1, and 1.0.-1.0, 24 genes). In total, we observed 87 genes (40%) corresponding to a TSG profile. All these results are interesting since they suggest that a large number of TSG are deactivated quite rapidly in prostate malignancy progression even since neoplasia. From the TSG profiles that were inactivated since PIN, we observed well-known TSGs such as UBE1L and ANXA1 (Additional file 1: Figure S2 and Figure 3). Supporting our findings, UBE1L has been implicating in growth suppression in lung cancer [34] and ANXA1 has been related to tumorigenesis and malignancy in prostate tumors [35].

**Table 2:** Significant profiles selected by BSM

**Profile**	**Genes**	**%**	**Folds**	**pValue**	**qValue**	**Type**
−1.-1.-1.1	10	4.7	110	4.55E-06	0.09129	Oncogene (Late)
−1.-1.1.1	1	0.5	Inf	0.00E+00	0.00100	Oncogene
−1.0.-1.1	2	0.9	10	1.01E-05	0.19985	Oncogene
−1.0.1.-1	25	11.6	1563	8.00E-07	0.01693	2 t
−1.0.1.0	29	13.5	199	7.30E-06	0.14565	Oncogene
−1.0.1.1	6	2.8	600	5.00E-07	0.01096	Oncogene
−1.1.0.1	2	0.9	42	2.40E-06	0.04871	Oncogene (Early)
−1.1.1.-1	13	6.0	Inf	0.00E+00	0.00100	2 t
−1.1.1.0	20	9.3	3333	3.00E-07	0.00699	Oncogene (Early)
−1.1.1.1	6	2.8	6000	5.00E-08	0.00200	Oncogene (Early)
1.-1.-1.-1	6	2.8	6000	5.00E-08	0.00200	TSG (Early)
1.-1.-1.0	16	7.4	2000	4.00E-07	0.00898	TSG (Early)
1.-1.-1.1	8	3.7	Inf	0.00E+00	0.00100	2 t
1.-1.0.-1	6	2.8	222	1.35E-06	0.02784	TSG (Early)
1.0.-1.-1	5	2.3	625	4.00E-07	0.00898	TSG
1.0.-1.0	19	8.8	184	5.15E-06	0.10314	TSG
1.0.-1.1	6	2.8	231	1.30E-06	0.02686	2 t
1.1.1.-1	35	16.3	412	4.25E-06	0.08548	TSG (Late) / MSG

Folds is the number of times the corresponding stage-state profile was observed in Tomlins dataset relative to the permuted dataset or “Inf” when not observed in permuted dataset.

There were 35 TSG profiles that change its state from 1 to −1 until metastases (1.1.1.-1 in Figure 3 and marked as late TSG in Figure 2 and shown in Additional file 1: Figure S3), which correspond to metastases suppressor genes (MSG) profiles [36,37]. From these 35 MSG-like profiles, ASAH1 and ITGAV were also included in a MSG-like profile in the Tomlins paper [5], which were present within the androgen signaling activity. TNFSF10 is as well a known TSG that induces apoptosis of tumor cells but not normal cells [38] and has been proposed as a metastases suppressor gene [39]. This supports our prediction for TNFSF10 as a MSG. We also found MLLT4 as a putative MSG though it has not been implicated in prostate cancer. MLLT4 has been suggested as a TSG since loss of expression was related to poor outcome in breast cancer [40]. Likewise, MIA3 has been found as a TSG in malignant melanoma where low expression was associated to cell migration [41]. This supports the prediction that MIA3 is a MSG in prostate cancer since deactivation in metastatic prostate would facilitate tumor movement.

We also observed 8 oncogene-like profiles corresponding to 76 genes, inactive in normal cells and active during tumor progression. From these, 3 oncogenes profiles representing 28 genes were activated since PIN (marked as early oncogene in Figure 2 and Table 1), 4 profiles containing 38 genes were activated from PCA, and 1 profile of 10 genes activated until metastasis (marked as late oncogene in Figure 2 and Table 1). From the 38 genes whose profiles were activated from PCA, we noted that the activation was always preceded by uncertainty in PIN suggesting that activation is transitory beginning in PIN rather than in PCA supporting heterogeneity of molecular states and changes in the activation state since PIN. From the oncogene-like profiles that are active since PIN (marked as early oncogenes in Figure 2 and Additional file 1: Figure S4), we observed TSPAN13 and E2F5. E2F5 is part of the E2F family that regulates cell cycle interacting with tumor suppressors [42] and has been implicated in malignancy in ovarian cancers [43]. TSPAN13 has been recently found overexpressed in prostate cancers and neoplasia respect to normal [44]. These results support our findings for oncogene-like profiles.

Surprisingly, we found 4 profiles representing a considerable number of genes involving two transitions (52 genes, marked as 2 t in Figure 2 and Additional file 1: Figure S5) where normal and metastases samples showed the same state either active or inactive. Tomlins *et al*. also found these transient states [5]. Between PIN and PCA, we found uncertainties but not transitions. The fact that benign and metastases share partially similar molecular states may have deep implications, that a unique line of clonal expansion is occurring where constrains imposed to tumor cells are changing during malignancy progression, or that proposed by Malins where primary tumor and metastatic cancer evolve concurrently [45]. The two-transitional state may explain and is supported by the increasing evidence where some genes can act as both oncogene or TSG [46-48].

We observed that not all samples within a stage have the same activation state for a given gene. For example, for Early TSG in Figure 2 and detailed in Additional file 1: Figure S2, not all samples within the PCA stage are clearly inactive (−1), there are some samples having an active state (+1, red dots), which could be considered as “noise” or “tumor heterogeneity”.

All these results suggest that our findings are likely to be relevant for prostate cancer progression.

### Comparison with other gene selection methods

Tomlins *et al*. used differential gene expression to analyze tumor progression, thus we compared whether the genes selected by BSM could also be selected using other equivalent procedures. Table 1 shows that the major overlap is 28% using the SAM method. From the top 215 genes selected by SAM (Additional file 2: Figure S6, 60 (28%) were also selected by BSM proposing an overlapped tendency (p < 1e-68). Nevertheless, functional analysis shown in Additional file 2: Table S4 and Additional file 2: Table S6 clearly show that the biological terms associated to SAM and BSM gene lists differ. For comparison, using top 2,726 genes from SAM (14% of the dataset at q=0) there were 21 out of 215 genes (9.8%) selected by BSM that were not included. At the same time, the BSM genes are obscured in the large list generated by SAM suggesting that BSM may be used to highlight genes with specific tumor progression profiles.

Interestingly, 11 of the 82 genes reported by Tomlins *et al*. were also selected by BSM suggesting a degree of similarity (p =< 1e-7). Given that the state-stage profiles found with BSM correspond to profiles shown by Tomlins *et al*., our method is contributing with 204 genes following similar profiles putatively involved in tumor progression.

An approach termed ‘barcode’ was proposed to classify several tissues selecting genes that were unexpectedly high expressed or low expressed in a specific tissue [21,49]. Since tumor stages can be seen as different cell types, Barcode may be used for a similar purpose than BSM. However, the barcode algorithm does not consider uncertainty in the threshold operation; it assumes and selects genes whose expression distribution has at least two clear peaks; it is designed and applied for detecting different tissues where gene expression changes drastically rather than detection of subtle differences within the same tissue; and it has been tested on Affymetrix technologies only. However, barcode does not consider tumor progression stages or studies; the gene expression for most of the genes are similar; and the preferred technology could be not Affymetrix. Moreover, we focused in selecting significant progression profiles such as those expected for TSG and oncogenes rather than selecting genes that are unexpectedly expressed or not expressed neither for classification purposes. All these arguments propose that barcode and BSM may select genes with different profiles. To test this, we used the 160 of the 215 selected genes having Entrez Id associated and fed into the barcode web tool (http://rafalab.jhsph.edu/barcode). The results in Additional file 2: Table S7 show that 47 (37%) genes out of the 127 genes included in Barcode database were associated to tissues (9 to prostate tumors and 38 to ‘many’ tissues). This result proves that BSM and Barcode select different genes. In addition, BSM can be applied to other microarrays technologies and for samples derived from the same tissue such as tumor progression.

In addition, we also tested the same gene selection methods to a prostate cancer dataset provided by the Memorial Sloan-Kettering Cancer Center [27]. As shown in (Additional file 1: Figure S9 and Additional file 2: Table S8), only the 11% of the genes selected by BSM are as well found by other methods.

Overall, these results suggest that information discovered by BSM is complementary to that obtained by other methods suggesting that BSM is a valuable tool for studying tumor progression.

### BSM progression signature as biomarker

Although BSM was intended for gene-discovery purposes, we wondered whether the profiles selected might be predictive of progression stage. Therefore, we also tested our 215 genes signature for classification measuring the accuracy of a nearest centroid classifier in a 10-fold-cross-validation scheme. Results are shown in Additional file 1: Figure S7. Our signature obtained 84% of accuracy while the first 215 genes ranked by SAM obtained 90%. The confusion matrix shown in Additional file 1: Figure S7. A suggests that our BSM signature confounds PIN and PCA. This is congruent since PIN and PCA differ mainly in profiles where PIN is uncertain and only one significant gene where the activation state was opposite between PIN and PCA. This indicates that BSM profiles barely distinguish between PIN and PCA. Nevertheless, we wondered whether a subset of the BSM signature might be more predictive. Using multivariate searches we have developed [50,51], we found a 13-genes model predicting correctly 94% of samples (Additional file 1: Figure S8). These results suggest that the BSM signature may also carry predictive information that can be used to distinguish tumor progression and therefore may be related to clinical outcome. To test this, we challenged the 215 genes signature in two prostate cancer datasets where outcome information is available and the majority of these genes are also included. We used the Sboner dataset [9] related to survival time after diagnosis and the Memorial Sloan-Kettering prostate cancer dataset [27] related to time to recurrence after radical prostatectomy. We used 75 of the 215 genes that are included in both datasets (Additional file 2: Table S10) in a Cox model and also the genes present in the 13-gene signature. We employed the risk score (fitted coefficients multiplied by gene expression) to generate two risk groups splitting the risk score by the median. The results shown in Additional file 1: Figure S10 indicate that the signature indeed is predictive of clinical outcome in the two datasets used. Therefore, the BSM signature is predictive of clinical outcome and can be valuable as a biomarker.

## Conclusions

We showed that simple binary states estimated to samples and then samples grouped into stages can identify genes putative related to tumor progression, which was also supported by biomedical literature. We showed that the profile of selected genes is similar to profiles expected for oncogenes, tumor suppressor genes and metastases suppressor genes and that these genes can be used for prediction purposes. In addition, we found genes with two changes in the activation state that require further investigation. We also demonstrated that our binary state model discovers genes not found by other methods. We conclude that the binary state model proposed here is a valuable tool to analyze tumor progression from gene expression data.

## Competing interests

All authors declare no competing interests.

## Authors’ contributions

EM collected data, implemented computational code, and drafted the manuscript. VT design and supervised the analysis, and edited the manuscript. Both authors read and authorized the final manuscript.

## Supplementary Material

Additional file 1 http://bioinformatica.mty.itesm.mx/BSM**. Figure S1.** Heat map representation of the 180 positive synthetic genes and corresponding state-stage profile. **Figure S2.** Heat map from the list of genes with a TSG profile of 1.-1.-1.0 and 1.-1.-1.-1 . **Figure S3.** Heat map from the list of genes with a TSG profiles of 1.1.1.-1 corresponding to MSG. **Figure S4.** Heat map from the list of genes with an Oncogene profile activated since PIN. **Figure S5.** Heat map from the list of genes with 2 t profiles. **Figure S6.** Comparison of genes selected by SAM. **Figure S7.** Predictive BSM and SAM signatures per sample. **Figure S8.** Multivariate-selected signature. 13 genes selected are shown in left. **Figure S9.** Heat map representation of the 360 genes selected in the MSKCC prostate dataset. **Figure S10.** Kaplan-Meier plots of the BSM signature tested in two prostate cancer datasets. Click here for file

Additional file 2: Table S1 Simulation results across methods. **Table S2.** Number of false assigned genes (NF) for each parameter combination. **Table S3.** Stage-State profile frequency and significance estimation. **Table S4.** Significant genes selected by BSM. **Table S5.** Functional annotation for genes selected by BSM. **Table S6.** Functional annotation for genes selected by SAM. **Table S7.** Association of BSM selected genes according to Barcode tool. **Table S8.** Comparison of genes selected by other methods in the MSKCC prostate dataset. **Table S9.** Comparison of genes selected by other methods in the Tomlins dataset without rescaling (no uniformization). **Table S10.** List of 75 genes used to generate survival curves. Click here for file
