# Supplementary material for: Modelling gene expression profiles related to prostate tumor progression using binary states
Source: Theor Biol Med Model. 2013 May 31;10:37. doi: 10.1186/1742-4682-10-37 (PMC3691825; doi:10.1186/1742-4682-10-37)
Supplement: Additional file 1 — http://bioinformatica.mty.itesm.mx/BSM. Figure S1. Heat map representation of the 180 positive synthetic genes and corresponding state-stage profile. Figure S2. Heat map from the list of genes with a TSG profile of 1.-1.-1.0 and 1.-1.-1.-1 . Figure S3. Heat map from the list of genes with a TSG profiles of 1.1.1.-1 corresponding to MSG. Figure S4. Heat map from the list of genes with an Oncogene profile activated since PIN. Figure S5. Heat map from the list of genes with 2 t profiles. Figure S6. Comparison of genes selected by SAM. Figure S7. Predictive BSM and SAM signatures per sample. Figure S8. Multivariate-selected signature. 13 genes selected are shown in left. Figure S9. Heat map representation of the 360 genes selected in the MSKCC prostate dataset. Figure S10. Kaplan-Meier plots of the BSM signature tested in two prostate cancer datasets. [file 1742-4682-10-37-S1.docx]

SUPPLEMENTARY DATA

Modelling gene expression profiles related to prostate tumor progression using binary states

Emmanuel Martinez-Ledesma and Victor Trevino

Tecnológico de Monterrey, Cátedra de Bioinformática, Monterrey, Nuevo León, México, 64849

# Supplementary Figures


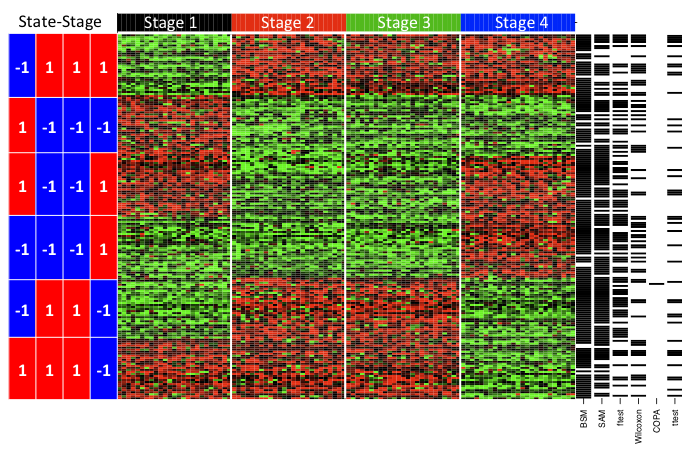


**Fig. S1**. Heat map representation of the 180 positive synthetic genes and corresponding state-stage profile. Brighter green, black, and red represent values from to 0, 0.5, and 1 respectively. Values between are represented by corresponding color gradient. The right panel shows the genes that were recovered (black) across methods.


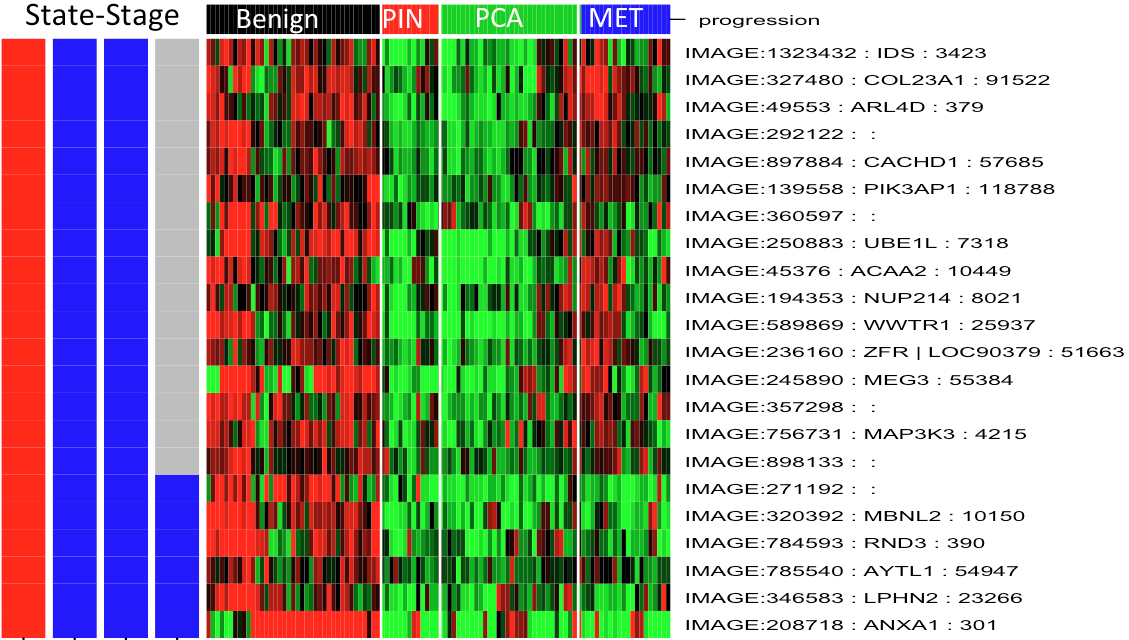


**Fig. S2**. Heat map from the list of genes with a TSG profile of 1.-1.-1.0 and 1.-1.-1.-1 . Stage-State is represented by red=1, blue=-1, and gray=0 in the same order than the heat map (Benign, PIN, PCA, Met). Gene labels contain clone id, gene symbol, and entrez number. Brighter green, black, and red represent values from to 0, 0.5, and 1 respectively. Values between are represented by corresponding color gradient.


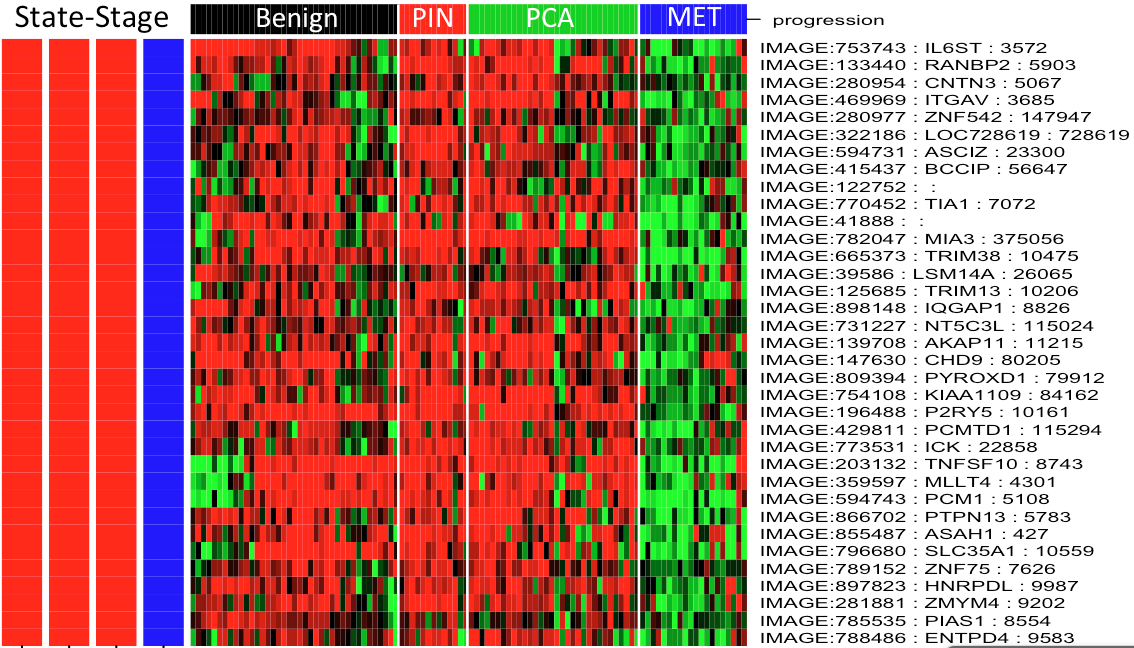


**Fig. S3**. Heat map from the list of genes with a TSG profiles of 1.1.1.-1 corresponding to MSG. Stage-State is represented by red=1, blue=-1, and gray=0 in the same order than heat map (Benign, PIN, PCA, Met). Gene labels contain clone id, gene symbol, and entrez number. Brighter green, black, and red represent values from to 0, 0.5, and 1 respectively. Values between are represented by corresponding color gradient.


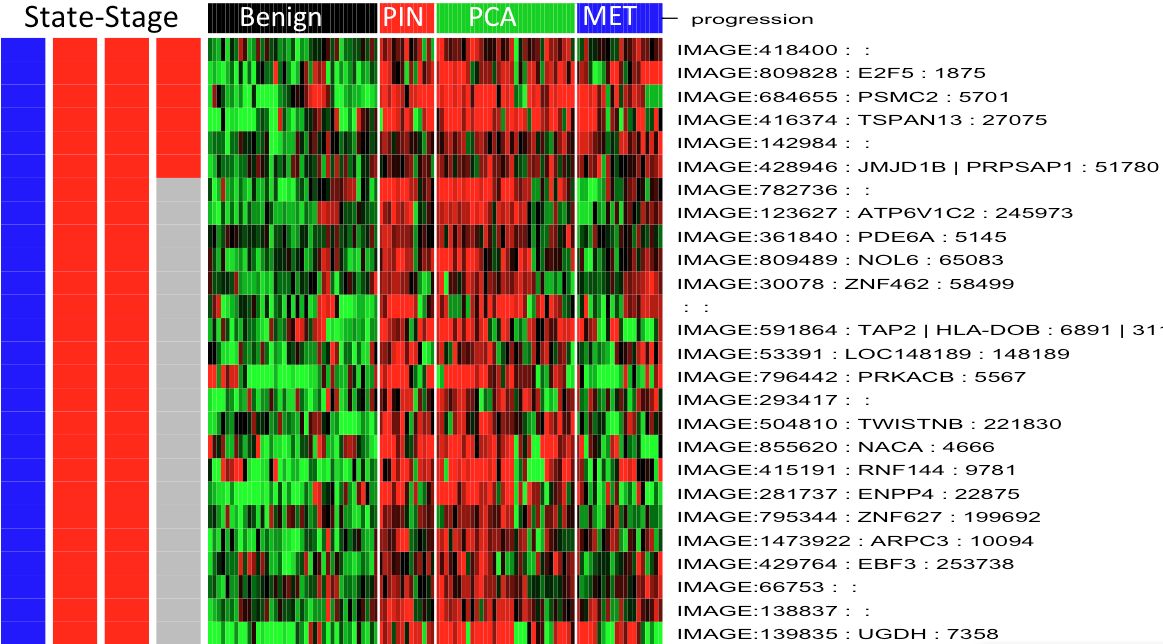


**Fig. S4**. Heat map from the list of genes with an Oncogene profile activated since PIN. Stage-State is represented by red=1, blue=-1, and gray=0 in the same order than heat map (Benign, PIN, PCA, Met). Gene labels contain clone id, gene symbol, and entrez number. Brighter green, black, and red represent values from to 0, 0.5, and 1 respectively. Values between are represented by corresponding color gradient.


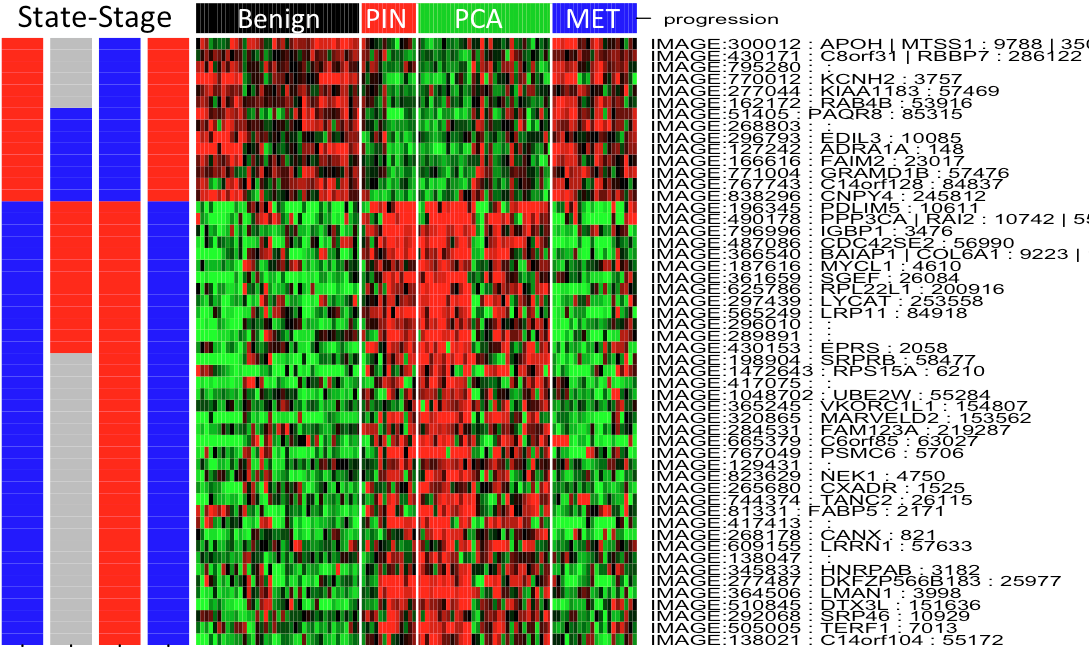


**Fig. S5** Heat map from the list of genes with 2t profiles. Stage-State is represented by red=1, blue=-1, and gray=0 in the same order than heat map (Benign, PIN, PCA, Met). Gene labels contain clone id, gene symbol, and entrez number. Brighter green, black, and red represent values from to 0, 0.5, and 1 respectively. Values between are represented by corresponding color gradient.

**
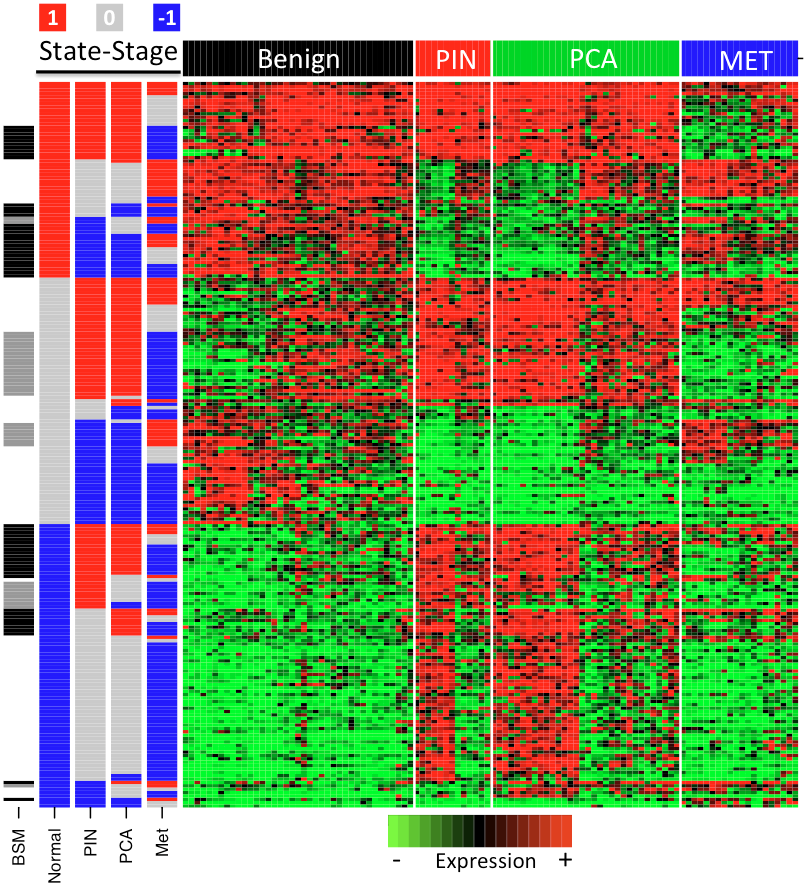
**

**Fig. S6**. Comparison of genes selected by SAM. State-Stages “signatures” of genes selected by SAM whose profile was estimated by BSM. State-stages are represented as active=1 in red, inactive=-1 in blue, or uncertain=0 in gray. Stages are indicated. Samples are shown in columns whereas genes are ordered by stage-state profile in vertical. Expression values ranges from 0 to 1 corresponding to various levels of colors from green to black then to red. Rank given by BSM is shown for comparison (black represents ranks from 1 to 215 which were selected in the main paper, dark gray represent ranks to 500, and white represents ranks larger than 2000, there were no BSM genes ranked between 500 and 2000). Clearly, interesting progression profiles are selected also by BSM.


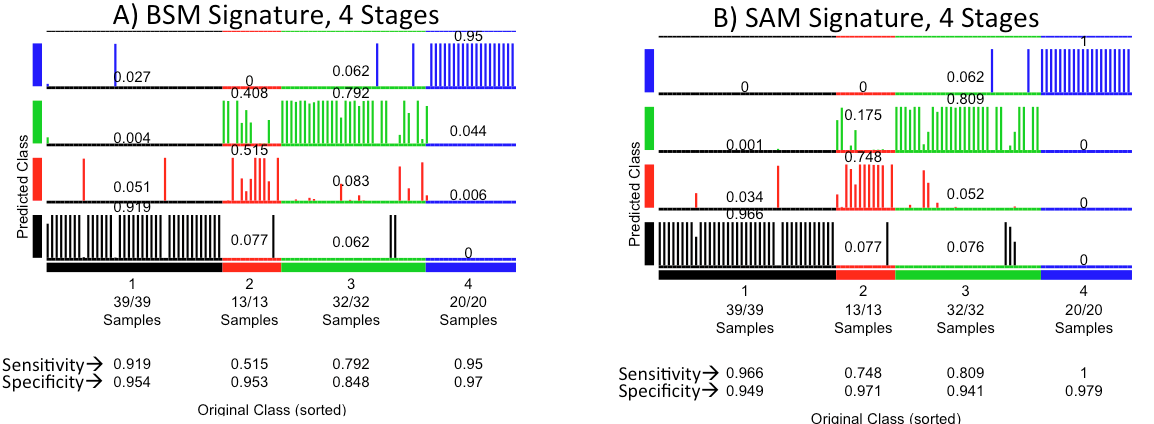


**Fig. S7.** Predictive BSM and SAM signatures per sample. Samples and stages are represented in horizontal axis as ‘Original Class’, 1=Black=Benign, 2=Red=PIN, 3=Green=PCA, 4=Blue=Met. Prediction stage is show in vertical as ‘Predicted Class’. Numbers represent fractions of prediction per stage. Overall sensitivity and specificity per stage is shown. Overall accuracy for BSM is estimated by Acc=(.919*39+.515*13+.792*32+.95*20)/(39+13+32+20)=0.8353 whereas for SAM Acc= (.966*39+.748*13+.809*32+1*20)/(39+13+32+20)=0.8969.


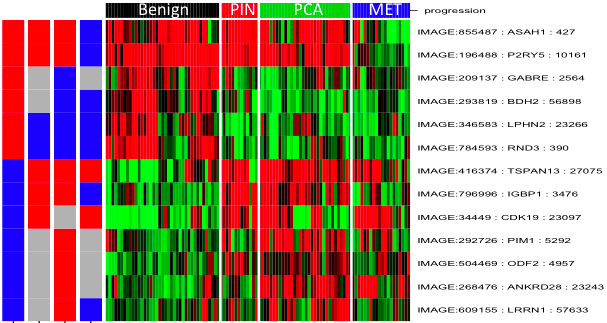

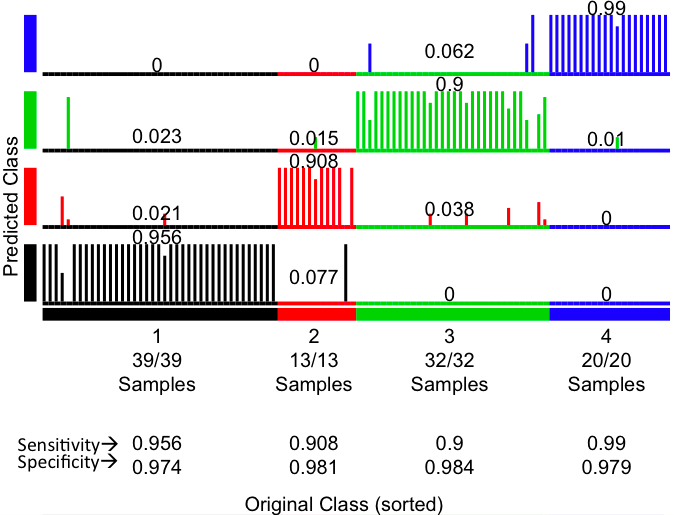


**Fig. S8.** Multivariate-selected signature. 13 genes selected are shown in left. Confusion matrix in right. Acc=(0.956*39+0.908*13+0.9*32+0.99*20)/ (39+13+32+20)=0.94. Accuracy estimated in test data for 30 random splits in 66% for training and 34% for testing.

**Fig. S9.** Heat map representation of the 360 genes selected in the MSKCC prostate dataset. Brighter green, black, and red represent values from to 0, 0.5, and 1 respectively in color gradient. The right panel shows the genes that were recovered (black) across methods.

**Fig. S10**. Kaplan-Meier plots of the BSM signature tested in two prostate cancer datasets. Figures show the dataset and clinical outcome. A Cox model was fit using the genes found in the dataset. A risk score was estimated using the Cox fitting and samples were split by the risk score median to generate both risk groups (green and red). Numbers in colors in x-axis represent the number of patients not presenting the event at corresponding time. Censored observations are represented by a “+” sign. P-value correspond to a log-rank test for the equality of survival curves. The 6-genes signature include TSPAN13, RND3, IGBP1, PIM1, ASAH1, and GABRE. The 13-gene signature include TSPAN13, RND3, IGBP1, BDH2, ANKRD28, ODF2, P2RY5, LRRN1, LPHN2, PIM1, CDK19, ASAH1, and GABRE.

# Supplementary Tables

Supplementary Tables S1 to Supplementary Table S7 are included as an Excel file in Bioinformatics Journal and in our website <http://bioinformatica.mty.itesm.mx/?q=node/86>.

Table S1. Results of the simulation experiment.

Table S2. Number of non-false assigned genes (NF) for each parameter combination.

Table S3. Stage-State profile frequency and significance estimation.

Table S4. Significant genes selected by BSM.

Table S5. Functional annotation for genes selected by BSM.

Table S6. Functional annotation for genes selected by SAM.

Table S7. Association of BSM selected genes according to Barcode tool.

Table S8. Comparison of genes selected by other methods in the MSKCC prostate dataset.

Table S9. Comparison of genes selected by other methods in the Tomlins dataset without rescaling (no uniformization).

Table S10. List of 75 genes used to generate survival curves.

# Algorithm for the BSM procedure:

1. Estimation of the best BSM parameters
   1. For each parameter combination (u, t, mp)
      1. Estimate BSM to the Gene Expression Dataset
      2. Estimate BSM to 100 Random Datasets
      3. Estimate “False Defined Genes” to i and ii.
   2. Select the u, t, mp parameters that minimized the “False Defined Genes” in 1A
2. Estimate the sample-stage profile
   1. Perform BSM on the Gene Expression Dataset using the parameters u, t, mp from 1B.
   2. Estimate the sample-stage profile by concatenating the 1’s, 0’s, and -1’s representing the activation state per stage.
3. Estimate the significance of the sample-stage profile
   1. For at least 1,000 times = Perm
      1. Generate a Permutated dataset by permutation of the sample stage labels per gene.
      2. Estimate BSM to Permutated dataset using the same u, t, and mp parameters from 1B.
      3. Estimate the sample-stage profile as in 2B
   2. Count the number of times each sample-stage profile is observed in all permutated datasets
   3. Estimate the significance of the observed sample-stage profile in the Gene Expression Dataset by (1+count of sample-stage profile) / (total permutation genes) = (1 + 3B) / (Perm * Dataset size in Genes).
   4. Correct the significance of the profile by a FDR approach.

# Pseudo-code for the BSM procedure

D =dataset composed by n genes and m samples

P= progression stages of length m

//Parameters

mp = {0.5, 0.6, 0.7, 0.8}

t = {0.3, 0.4, 0.5, 0.6, 0.7}

u = {0, 0.25, 0.5, 0.75, 1}

bmatrix = matrix with length(mp)*length(t)*length(u) rows and (m + 1) columns

rmatrix = matrix with length(mp)*length(t)*length(u) rows and (m + 1) columns

**foreach (mp)**

**foreach(t)**

**foreach(u)**

apply BSM to D with parameters mp[x], t[y], u[z]

bmatrix = count how many genes are defined in 0 to m stages for each parameter combination

**for(i from 1 to 100)**

RD = random generated dataset composed by n genes and m samples

**foreach (mp)**

**foreach(t)**

**foreach(u)**

apply BSM to RD with parameters mp[x], t[y], u[z]

rmatrix= count how many genes are defined in 0 to m stages for each parameter combination

//For example if a dataset has 4 stages, bmatrix and rmatrix might look like this

// #number of stages 0 1 2 3 4

//"mp=0.7,t=0.5,u=0" 1558159 409453 31585 698 5

//"mp=0.7,t=0.5,u=0.25" 1940231 59206 463 0 0

//"mp=0.7,t=0.5,u=0.5" 1995177 4722 1 0 0

//"mp=0.7,t=0.5,u=0.75" 1999679 221 0 0 0

//"mp=0.7,t=0.5,u=1" 1999896 4 0 0 0

**search Parameters (bmatrix, rmatrix/100)**

auxmat = matrix with equal rows as bmatrix or rmatrix and one more column than bmatrix or rmatrix

**for(i from 1 to number of rows of bmatrix)**

**for(j from number of columns of bmatrix to 1)**

numer = sum(rmatrix[i, j : number of columns of bmatrix])

denom = sum(bmatrix[i, j : number of columns of bmatrix])

auxmat[i,j] = **if (denom = 0)** 1 **else** numer/denom

**if (j = 1)**

auxmat[i , number of columns of auxmat] = sum[(1 - auxmat[i, 2 : number of columns of bmatrix]) * bmatrix[i, 2 : number of columns of bmatrix]]

// Here * is applied element by element

// The last column from auxmat represents a score of the Estimated Non-False

// genes (NF)

params = choose the params where max (NF)

**for (i from 1 to 1000)**

PD = generate an artifical data set by permuting genes and class labels.

apply BSM to PD using the parameters in params

table_profiles = obtain the frequency for each gene profile

//table_profiles might look like this:

// Gene profiles 1.1.1.-1 1.0.1.-1 1.-1.1.-1

// Frequency 2687 1534 865

D_profiles = apply BSM to D using the parameters in params

**foreach( gene profile in D_profiles)**

Obtain p-value diving the frequency of the gene profile in table_profiles / total frequency of table_profiles

qbals = adjust p-values
